# Assessing Non-Photosynthetic Cropland Biomass from Spaceborne Hyperspectral Imagery

**DOI:** 10.3390/rs13224711

**Published:** 2021-11-21

**Authors:** Katja Berger, Tobias Hank, Andrej Halabuk, Juan Pablo Rivera-Caicedo, Matthias Wocher, Matej Mojses, Katarina Gerhátová, Giulia Tagliabue, Miguel Morata Dolz, Ana Belen Pascual Venteo, Jochem Verrelst

**Affiliations:** 1 Department of Geography, Ludwig-Maximilians-Universitat Munchen (LMU), Luisenstr. 37, 80333 Munich, Germany; 2 Institute of Landscape Ecology, Slovak Academy of Sciences, Branch Nitra, 949 01 Nitra, Slovakia; 3 Secretary of Research and Postgraduate, CONACYT-UAN, Tepic 63155, Mexico; 4 Remote Sensing of Environmental Dynamics Lab, University Milano-Bicocca, 20126 Milano, Italy; 5 Image Processing Laboratory (IPL), Parc Cientific, Universitat de Valencia, 46980 Paterna, Spain

**Keywords:** PRISMA, CHIME, NPV, Gaussian process regression, hybrid retrieval, active learning, PCA, PROSAIL-PRO

## Abstract

Non-photosynthetic vegetation (NPV) biomass has been identified as a priority variable for upcoming spaceborne imaging spectroscopy missions, calling for a quantitative estimation of lignocellulosic plant material as opposed to the sole indication of surface coverage. Therefore, we propose a hybrid model for the retrieval of non-photosynthetic cropland biomass. The workflow included coupling the leaf optical model PROSPECT-PRO with the canopy reflectance model 4SAIL, which allowed us to simulate NPV biomass from carbon-based constituents (CBC) and leaf area index (LAI). PROSAIL-PRO provided a training database for a Gaussian process regression (GPR) algorithm, simulating a wide range of non-photosynthetic vegetation states. Active learning was employed to reduce and optimize the training data set. In addition, we applied spectral dimensionality reduction to condense essential information of non-photosynthetic signals. The resulting NPV-GPR model was successfully validated against soybean field data with normalized root mean square error (nRMSE) of 13.4% and a coefficient of determination (R^2^) of 0.85. To demonstrate mapping capability, the NPV-GPR model was tested on a PRISMA hyperspectral image acquired over agricultural areas in the North of Munich, Germany. Reliable estimates were mainly achieved over senescent vegetation areas as suggested by model uncertainties. The proposed workflow is the first step towards the quantification of non-photosynthetic cropland biomass as a next-generation product from near-term operational missions, such as CHIME.

## 1 Introduction

Quantification and knowledge of non-photosynthetic vegetation (NPV) or vegetation brownness [1] are crucial in all terrestrial ecosystems [2]. NPV includes those plant elements and organs that do not or no longer perform photosynthesis, such as dead vegetation, plant litter, or senescent foliage, branches and stem tissues [3]. NPV can be also plants in dormant status, as typical for some grasses. The actual amount of NPV on terrestrial vegetation strongly affects carbon and nutrient cycling, erosion, and risk of fires [4,5]. Knowledge of NPV is further important for monitoring seasonal, annual, and long-term changes of terrestrial vegetation. For agricultural applications, typically two forms of NPV are specifically of interest [3]. At first, we refer to crops at late mature growth stages, e., cropland biomass. Senescence in cultivated land can indicate mortality caused by disturbance events, such as droughts. As a second important variable, we consider crop residues (CR), i.e., the plant material left on the fields after the crop has been harvested [6]. CRs influence soil temperature and humidity, and thus the rate of chemical and biological reactions in the soil [7]. Moreover, CRs counteract soil erosion through wind and water, are a defence against weed growth [8], improve soil aeration [9], and enhance soil organic carbon fluxes [10,11]. Hence, retention of CR on the fields is an essential agricultural conservation practice based on minimum tillage [12].

NPV is mainly composed of cellulose, lignin and hemicellulose, which together form the "lignocellulosic biomass" [13], making up approximately 90% of plant dry matter content [14]. The polymer cellulose is considered the main constituent of plant cell walls and is one of the most abundant biomaterials on Earth [15]. Lignin, on the other hand, belongs to the polyphenolic compounds of plants. It is a complex, hydrophobic molecule of aromatic nature [16,17]. Along with some other polysaccharides, cellulose and lignin are intertwined in a complex way in plant cell walls [14]: whereas cellulose is bound by plant leaves for the wall of parenchyma cells, lignin is part of the secondary cell walls of vascular fibers. Typically these two main compounds are lumped together and termed "cellulose-lignin" [6] or "ligno-cellulose" [18]. Being an essential part of the total organic carbon within the biosphere, the spatiotemporal knowledge of the lignocellulosic plant material (CR or NPV) would increase our understanding of carbon fluxes, soil carbon loss or drought effects. Thus, this knowledge may help to optimize the role of agricultural landscapes in the global carbon cycle. However, the quantification of non-photosynthetic cropland biomass is missing at appropriate spatial (and temporal) scales and over large areas. To achieve this, efficient and accurate methods are required to quantify NPV biomass from individual fields to wide cultivated areas [6].

The main problem in the quantification of NPV is its discrimination from different types of bare soils, in particular in the visible and near-infrared spectral domains. NPV is spectrally different from soils in the shortwave infrared (SWIR, appr. 1300-2500 nm), which is mainly caused by absorption of non-pigmented organic molecules, primarily lignin and cellulose [19]. According to Daughtry et al. [6], in particular the broad absorption feature near 2100 nm is evident in the reflectance spectra of CR, but absent in soil spectra [19–21]. Two more important absorption features are located close to 1730 nm and 2300 nm, primarily being associated with lignin and cellulose, respectively [18,22]. Broadband multispectral sensors are generally limited in their ability to detect NPV because of missing narrow bands required to resolve these features [23,24]. By separation of leaf area index (LAI) from green and senescent vegetation, Amin et al. [25] demonstrated the first streamlined production of brown LAI from Sentinel-2 imagery. Nonetheless, the authors also confirmed the challenge of quantifying senescent plant material from multispectral data due to the spectral similarity between brown LAI and bare soils. Imaging spectrometers, commonly referred to as hyperspectral sensors, can provide the required contiguous bands with 5-10 nm full-width half maximum (FWHM) in the SWIR to detect NPV [26] and distinguish it from bare soils. Hence, with the new fleet of spaceborne imaging spectrometers [27] capable of resolving the lignocellulose absorption features, new opportunities open up for the development of efficient models to quantify NPV biomass. These missions include the recently launched PRecursore IperSpettrale della Missione Applicativa (PRISMA) [28], the forthcoming Environmental Mapping and Analysis Program (EnMAP) [29], the planned NASA Surface Biology and Geology observing system (SBG) [30] and the Copernicus Hyperspectral Imaging Mission for the Environment (CHIME) [31]. The CHIME's mission advisory group (MAG) identified a group of high priority vegetation and soil variables to be provided as operational products. Among others, these include lignin and cellulose to quantify the amount of NPV biomass [32].

In the past decade, the focus was mainly on the quantification of crop residue or NPV coverage (in %) from remote sensing data (e.g., [33]). Hereby, applied methods ranged from empirical algorithms (e.g., crop residue indices), over classification [34], to spectral unmixing [11], spectral angle methods [35] and spectral mixture analysis (SMA) [36,37].

In contrast to CR or NPV coverage, the estimation of NPV biomass has been only rarely performed [38], for instance by Numata et al. [39] for grazed pastureland and by Ren and Zhou [40] for desert steppe.

However, the quantification of lignocellulosic biomass is important for diverse agricultural disciplines. Among those, breeding research aims to understand the role of lignin content in animal nutrition and biofuel productions [41]. A recent review summarizes the role of lignin in view of crops susceptibility towards biotic and abiotic stresses, important for agroindustrial processes [42]. For instance, lignocellulosic plant biomass can be converted to liquid fuels within thermochemical energy conversion processes [43]. Moreover, according to the review study of Li and Guo [38], NPV biomass presents an ideal indicator of NPV abundance and carbon source [44]. All in all, this calls for quantitative estimation of NPV or lignocellulosic plant biomass using appropriate units (i.e., g/m^2^ or kg/ha), opposed to the rather rough estimation of surface coverage in percentage. In respect to retrieval methods, exclusively parametric regressions have been investigated (e.g., [39,40,45]). As most popular index to quantify NPV, the Cellulose Absorption Index (CAI) has been widely explored [20,46]. Other approaches include the Green Brown Vegetation Index (GBVI) by Delegido et al. [47] or the dry matter index proposed by Romero et al. [48]. Yet, there is a lack of studies exploring physically-based approaches including radiative transfer models (RTMs) and machine learning (ML) regression algorithms, being increasingly exploited for the retrieval of diverse vegetation variables [49]. Recently, progress was made to improve the representation of NPV in leaf optical properties models. For instance, the PROSPECT-PRO model [50] introduced the carbon-based constituents (CBC), encompassing cellulose, lignin, hemicellulose, starch and sugars. Feret et al. [50] demonstrated that CBC can be estimated from optical properties of both fresh and dry foliage. To upscale the leaf optical properties to the canopy level, PROSPECT-PRO can be coupled with the Scatter- ingby Arbitrarily Inclined Leaves model (4SAIL) [51], to PROSAIL-PRO [52]. Such RTMs can simulate a wide range of vegetation states; thereby replacing the need of in situ field measurements to generate training data sets for ML algorithms. Hence, combining RTMs with ML led to the promising hybrid methods [49,53], which offer an efficient and versatile balance between physics-awareness and data driven approaches [54]. In the past, retrieval of crop properties has been mainly based on parametric regressions, which denominate empirical relationships between spectral observations (or vegetation indices) and in situ measured variables [53,55]. These rather simple methods can be easily implemented and have the advantage of being computationally fast. Besides a clear under-exploitation of available spectral information content, these methods miss a retrieval quality indicator in the form of per-pixel uncertainty and thus are fundamentally limited in their genericity and transferability [49,56,57]. The usage of physically-based inversion methods [58,59] may overcome some of these drawbacks, but they are usually computationally expensive, limiting their applicability for hyperspectral data streams. Therefore, hybrid methods may present an ideal path towards generic, physically-based and efficient derivation of vegetation or crop properties from imaging spectroscopy data [49,52,60,61]. In respect to suitable ML algorithms for this task, nonparametric kernel methods and in particular Bayesian approaches of Gaussian processes regression (GPR) [62] have demonstrated to outperform other ML algorithms. This competitiveness is due to a number of theoretical and practical advantages of GPR algorithms, such as the design of appropriate covariance functions, allowing to include prior knowledge about the signal characteristics [62,63]. Additionally, with their property to deliver predictive variance (i.e., uncertainty intervals), GPR models provide important information about their retrieval quality and thus transferability. This distinct feature is not shared by any other machine learning regression approach [64]. Moreover, quantifying variable-associated uncertainty is a pre-requisite to ingest remote sensing products in higher-level processing models [63].

In the last few years, hybrid methods have been proposed and implemented for deriving high priority variables for CHIME and other future missions [32,60,65,66]. Recently, two new features were introduced into hybrid models to reduce and optimize the dimensionality of the training data sets: (1) active learning (AL) and (2) feature transformation. These two key steps led to successful mapping of landscape canopy nitrogen content (CNC) from PRISMA hyperspectral data [60]. In view of the prioritization of those specific variables by near-term operational missions, it remains to be investigated if such advanced models could also be used for quantification and mapping of non-photosynthetic cropland biomass. Therefore, this study aimed at implementing a hybrid workflow for the retrieval of non-photosynthetic cropland biomass from imaging spectroscopy data. To achieve this, an experimental case study was performed using radiative transfer modeling combined with machine learning regression approaches. Overall, with our study, we aim to give inspirations towards new possibilities for routinely mapping of NPV biomass from operational spaceborne imaging spectroscopy missions, such as CHIME.

## 2 Materials and Methods

## 2.1 Design of the Workflow

**Figure 1:**
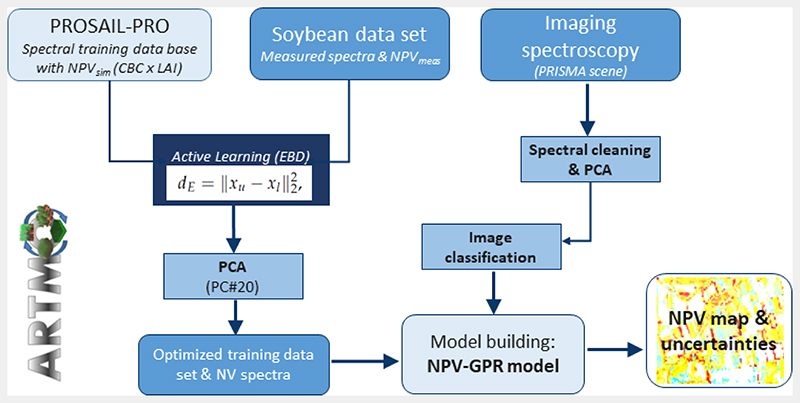
Schematic workflow for NPV mapping.

The essence of hybrid strategies for vegetation traits retrieval is that a ML regression algorithm is trained by simulated data coming from coupled leaf-canopy RTMs. For our study, we adopted a hybrid method as proposed and successfully tested by prior works for diverse vegetation traits [32,60]. Figure 1 outlines schematically the workflow consisting of six main steps, which will be described in detail in the next sub-chapters:

1. Generating a training data base with PROSAIL-PRO,
2. Applying active learning methods to reduce and optimize the training data set,
3. Adding non-vegetated (NV) spectra,
4. Reducing dimensionality of simulated and measured spectra in the SWIR domain,
5. Classifying of the satellite scene to identify croplands and bare soils,
6. Processing the scene over surfaces of interest with the NPV-GPR retrieval model to estimate non-photosynthetic cropland biomass.

## 2.2 Modeling Approaches

**Figure 2:**
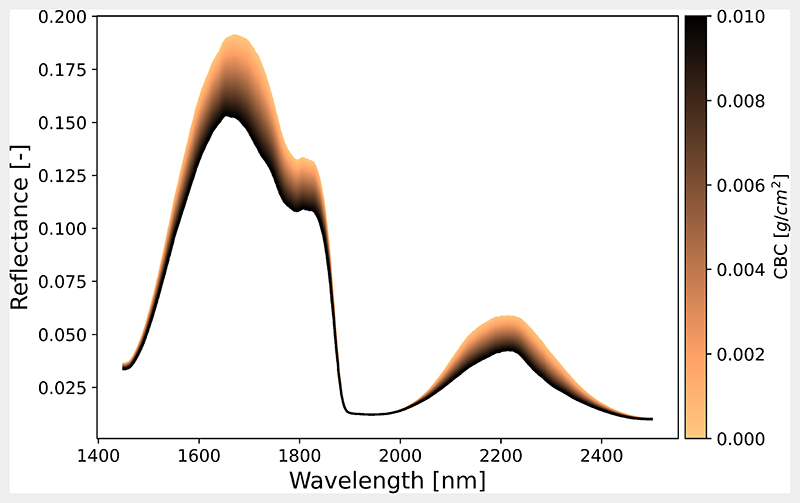
Local sensitivity analysis of carbon-based constituents in the SWIR domain using the Interactive Visualization of Vegetation Reflectance Models (IVVRM) tool of the EnMAP-Box Agri- Apps. CBC was ranged between 0-0.01 and all other variables were fixed to standard values.

To obtain non-photosynthetic vegetation traits of a cropped surface from imaging spectroscopy data, one requires a model that derives these traits from the spectral observations. In this study, we explored the carbon-based constituents variable of the PROSPECT-PRO leaf optical properties model [50]. To visualize the sensitivity of CBC on a canopy reflectance spectrum, we used the Interactive Visualization of Vegetation Reflectance Models (IVVRM) toolbox embedded in the Agricultural Applications (Agri-Apps) of the EnMAP- Box [67]. The IVVRM tool enables to perform local sensitivity analysis for a range of RTMs. Hereby, the impact of diverse model leaf biochemical and canopy structural input parameters is assessed on the overall spectral signal. In contrast to global sensitivity analysis (GSA), this tool allows only the one-factor-at-a-time (OAT) approach: one calculates the effect of the variation of a model parameter (factor) when all others are kept constant at nominal values [68]. Figure 2 illustrates the impact of CBC in the SWIR, which is particularly apparent in the spectral region of 1600-1800 nm and 2100-2300 nm. Documentation and installing instructions of the IVVRM toolbox within the EnMAP-Box Agri- Apps can be found at https://enmap-box-lmu-vegetation-apps.readthedocs.io/en/latest/, accessed on 10 November 2021.

To upscale CBC to the canopy level, PROSPECT-PRO was coupled with 4SAIL to PROSAIL-PRO. The combined model simulates spectral reflectance as a function of diverse biochemical and biophysical input parameters. The following ranges were set according to the experience of the authors and prior studies [52,56,60,61,65,69]: leaf water content *(C*_w_) from 0-0.02 cm, leaf structure parameter (N) from 1-2, leaf carotenoid content (C_xc_) from 0-15 pg/cm^2^, leaf anthocyanins content *(C_an_t*) from 0-2 pg/cm^2^, brown pigments (Q) from 0-0.5 pg/cm^2^, leaf protein content *(Cp*) from 0-0.0025 g/cm^2^, average leaf inclination angle (ALIA) from 30-70 degree, hot spot parameter from 0.01-0.5 (unitless), and the soil reflectance factor was varied between 0-1 to scale between two model-implemented soil spectra (dry and wet). Leaf chlorophyll content *(C_a_b*) was ranged from 0-20 pg/cm^2^ to account for mainly senescent plant organs. The key variable CBC was varied between 0-0.007 g/cm^2^ (mean 0.004 and standard deviation, SD: 0.001), and LAI from 0-4 m^2^/m^2^ (mean: 2.0 SD: 2.0). CBC and LAI were sampled with Gaussian distributions to realistically simulate variations of NPV biomass at mature and senescent growth stages. In this way, the variable sampling corresponded approximately to the measurement range over soybean crops (see Section 2.5). All the other variables were sampled uniformly to allow global applicability. Note that in particular, the pigment contents do not influence the simulations since only the SWIR domain is considered by subsequent processing steps.

NPV was modelled multiplying CBC and LAI. Finally, simulated "aboveground CBC content", denoted here as *NPV_S_i_m_,* in [g/m^2^], was added to the training data base. Overall, we generated a synthetic data set consisting of 1'000 different vegetation states and corresponding bidirectional canopy reflectance (step 1). This size was decided according to suggestions of previous studies [60,61,66].

## 2.3 Optimizing Spectral and Sampling Configurations

To achieve optimal performances within a hybrid workflow, the quality or representativeness, rather than the quantity of a training data set, is the key [61]. Therefore, we implemented AL techniques [70,71] to reduce and optimize the information content of the samples (step 2). Specifically, we decided for the Euclidian distance-based (EBD) *diversity* strategy [72], which, according to a recent survey, performed superior to most other methods in terms of accuracy and processing speed [61]. Starting with a randomly selected initial data set (N = 10), EBD selected those samples out of the simulated data pool which were most distant from the already included ones (see also Figure 1). At each iteration, the new sample was added when accuracy against the in situ data set (see Section 2.5) improved, which was evaluated by the root mean square error (RMSE). However, when the RMSE increased, the sample was ignored and the algorithm proceeded with the evaluation of the next sample. The whole process was repeated until all samples of the training data set were evaluated.

Subsequently, 24 NV spectra were extracted from the PRISMA scene and added to the training data base (step 3). These spectra covered all kinds of non-vegetated surfaces, including bare soils, water bodies and man-made surfaces. This measure avoids model failure due to unknown soil spectral signatures and assures that the model is applicable to the complete heterogeneous scene.

As next step (4), simulated (and measured) reflectance was transformed into 20 features using principal component analysis (PCA, [73]). With PCA, the spectral data are converted into a lower-dimensional feature space, still ensuring that the majority of the original information is preserved. PCA helps to identify features that are prevalent in most bands, but also those that cause a signal in some specific bands. In contrast to prior studies targeting the full optical range (400-2500 nm) to derive green (photosythetically active) vegetation traits [32,66], our analysis was restricted to the SWIR (1500-2500 nm), which allowed to focus on the ligno-cellulose absorption features. We decided for a total number of 20 principal components (PCs) following internal tests and previous studies [32,60,69]. Recently, this optimal number was confirmed by Morata et al. [74]. The authors suggested that using around 20 PCs is crucial to sufficiently represent the spectral variability, whereas the inclusion of more components failed in further error reduction. Moreover, 20 PCs are ideal to reliably represent the hyperspectral spectrum (input), keeping a compromise between the amount of exploited information and the computational cost of the model.

## 2.4 Machine Learning Regression Algorithms

The core algorithm within the hybrid scheme was based on GPR algorithms, which have proven excellent performances in multiple studies [49,75–79]. Furthermore, as GPR is based on a probabilistic treatment of regression problems, an analytical expression of the predictive uncertainty is provided along with final estimates [62]. Together with the high accuracy achieved with these algorithms, this specific characteristic renders GPR particularly attractive for solving regression problems within Earth observation (EO) data analysis: information of uncertainty in the model parameterization or input data can be used to assess the models transferability to other locations and times [80,81]. For our study, we adapted the formulation and equations from Rasmussen and Williams [62]. Moreover, extensive description of the GPR algorithms in the context of EO data analysis is provided in Camps-Valls et al. [63,82] and in Verrelst et al. [49,53]. Note that GPR algorithms typically can process only a few thousand samples within reasonable running time, as processing time rises exponentially with increasing size: for GPR, the calculation is based on the inversion of a N x N matrix, with N the number of simulations [62]. Nevertheless, these relatively small training data sets (here we use a size of 1'000) are well explored by these competitive kernel-based algorithms, which identify the most relevant bands (or components) and provide stable and accurate predictions for multiple EO estimation problems [83].

The final model for the estimation of non-photosynthetic cropland biomass is denoted here as NPV-GPR retrieval model.

## 2.5 Description of Data Set and Test Sites

**Figure 3:**
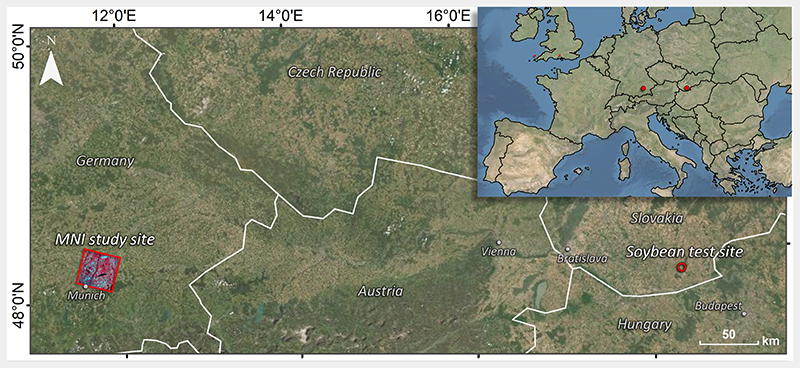
Location of the two study sites: PRISMA acquisition at MNI in Southern Germany and soybean sampling in Southern Slovakia.

For the present study, we explored a campaign data set from a highly variable soybean crop field, located in Southern Slovakia (47°55' N, 18°22' E), as illustrated in Figure 3.

For the analysis of this study, only measurements from mature crop growth stages were considered at two dates: 2 September 2020 with predominantly yellowish plants, and 16 September 2020 with solely brownish plant organs (see photographs in Berger et al. [65]). At several plots, hyperspectral canopy reflectance was measured with an ASD FieldSpec4 instrument. Subsequently, after each measurement, the full aboveground crop biomass (i.e., leaves, stalks and fruits) was cut, packed in bags and brought to the lab. The AM350 LAI meter was employed for organ area scanning to obtain measurements of total brown LAI (BAI, in m^2^/m^2^). In addition, dry mass per unit leaf area (DMb in g/cm^2^) was estimated based on the ratio of oven-dried leaf weight and respective leaf area. In total, 64 leaf samples were used for the estimation of *DMb* on both dates. In fact, leaf dry mass comprises mainly organic constituents, such as cellulose, lignin, starch, and sugar. Hence, we assumed CBC simulated by PROSPECT-PRO (as described in Section 2.2) as a good proxy for *DMb*. In order to be consistent with *NPV_S_i_m_* approximation at canopy level, *DMb* was multiplied by BAI, resulting in *NPV_meas_* Equation (1).

(1) $$\text{NDVI}=\frac{\left(\text{NIR}-\text{Red}\right)}{\left(\text{NIR}+\text{Red}\right)}$$

Finally, a total number of 14 measurements (mean: 55.9 g/m^2^, standard deviation: 31.0 g/m^2^, min: 15.7 g/m^2^ and max 111.7 g/m^2^) was available for validation and tuning of the model.

For mapping demonstration, a PRISMA image was acquired over the North of Munich, Germany, on 04/10/2020, covering the area of the Munich-North-Isar (MNI) campaigns (48° 16' N, 11°42' E), see also Figure 3. For a few years, MNI has been serving as validation core site for agricultural algorithms in the framework of the German EnMAP mission [52,60,84]. The PRISMA satellite carries a high-spectral resolution visible-near infrared (VNIR) to SWIR imaging spectrometer, able to capture images in 234 contiguous spectral bands, where 171 are located in the SWIR. The sensor provides a spectral resolution of less than 12 nm and a spatial resolution of 30 m [85]. For our purpose, a standard L2D PRISMA at-surface reflectance product was downloaded from the PRISMA ASI portal and pre-processed to obtain smooth spectra. At first, we used the findpeaks function of the R pracma package [86] to remove spikes occurring at diverse wavelengths along-track. For the actual image, we used a threshold of 0.018 for detecting peaks. In the next step, spectral regions providing too many noisy bands were systematically excluded. This procedure was based on a visual comparison against ground spectra, acquired during an Italian campaign using a field spectroradiometer (SR-4500; Spectral Evolution, Haverhill, MA, USA). Finally, we applied a spline smoothing interpolation with the SplineSmoothGapfilling function implemented in the R FieldSpectroscopyCC package [87]. This led to clean PRISMA spectra, except for the atmospheric water absorption located at 1350-1510 nm and 1795-2000 nm, which were finally removed. See also Figure 2 in Verrelst et al. [60] for a visual illustration of the procedure, applied to a PRISMA scene over the same region.

As step 5, a classification was applied to the scene to identify the surface types of interest. After a preliminary analysis with multiple classifiers, the standard nearest neighbor method proved to be most effective. Five classes were defined: man-made, water, forest-natural vegetation, crops and grasslands, and bare soils, with a total of 218 samples and a 3-k cross-validation strategy. As these classes are easily separable with hyperspectral data and a dimensionality reduction of 20 PCA components, it led to an overall accuracy of 98.1% (Kappa: 0.98). The resulting map served as mask for the non-photosynthetic biomass cropland mapping. Note that at this time of the year the majority of croplands is harvested and the fields are mainly covered by crop residues or grasses. Ultimately, the PRISMA scene was processed (step 6), applying the NPV-GPR retrieval model to the two classes of crops and grasslands, and bare soils.

The development of the hybrid model and subsequent mapping was done within the scientific Automated Radiative Transfer Models Operator (ARTMO, Verrelst et al. [88]) software framework. ARTMO includes the machine learning regression algorithm (MLRA) toolbox with an integrated active learning module [70] for retrieval applications. Multiple kinds of MLRAs, spectral dimensionality reduction and sampling strategies can be combined and applied. Moreover, a new machine learning classification toolbox (v1.00) has been developed within the ARTMO framework, which is demonstrated here for the first time.

## 3 Results

### 3.1 Optimization of Sampling

**Figure 4:**
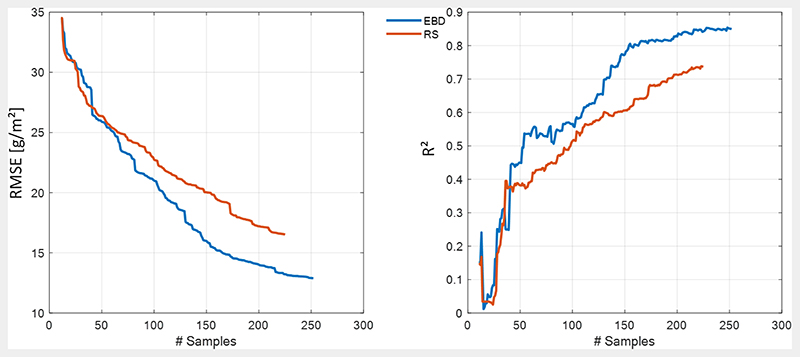
Goodness-of-fit statistics (left): RMSE and (right): R^2^ for non-photosynthetic cropland biomass retrieval applying the EBD method and random sampling (RS) on a PROSAIL-PRO simulated training database against in situ data.

In the first step, most informative and representative training samples were searched for by means of AL. Typically, training data sets of 1'000 simulations were built for the retrieval of diverse crop traits [52,66,80]. This, however, can lead to overly heavy matrices, being a strong limitation in the context of cloud processing, for instance. Thus, light retrieval models should be strived for to avoid memory issues for GPR algorithms. Figure 4 demonstrates the efficiency of the diversity EBD method for reducing and optimizing the training sample, using RMSE (left) and coefficient of determination (R^2^) (right) to describe the course of increasing accuracy. The usage of RMSE as criterion to keep or maintain a sample is reflected in its smoother convergence compared to R^2^. However, in general R^2^ follows the same pattern. Highest accuracy was obtained with the EBD method as opposed to random sampling (RS), reducing the RMSE from 35 to 12.9 and 17 [g/m^2^], respectively. Starting with 10 samples, the EBD sequence stopped at 252 samples, after all samples were evaluated, finally achieving R^2^ of 0.85 (R^2^ = 0.72 for RS).

### 3.2 Dimensionality Reduction

**Figure 5:**
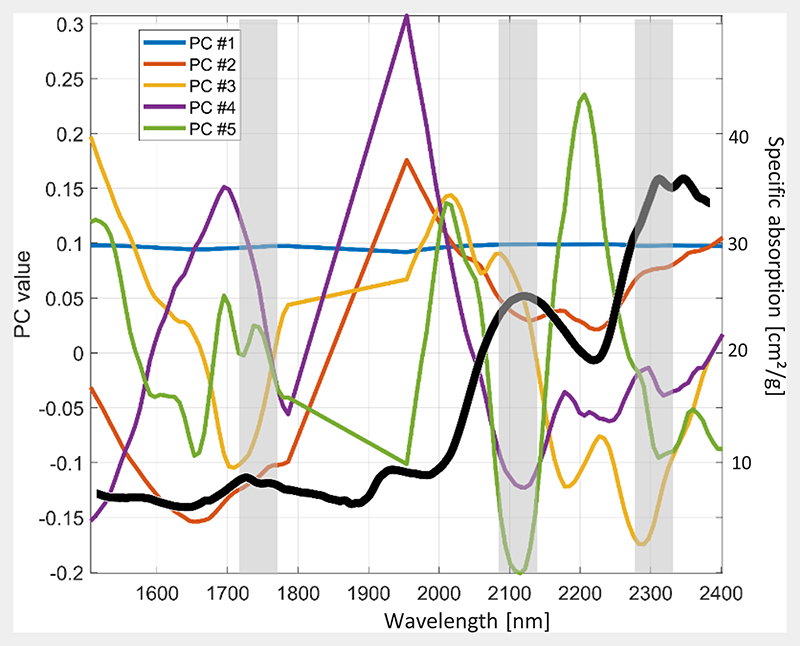
Principal component values (PC#1 to PC#5) as a function of wavelength, calculated from the simulated training data set. Specific absorption of CBC is given as a black line, and main absorption features of lignin and cellulose are indicated as grey bars.

Following the AL step, we continue with inspecting the information content compressed into the components. Figure 5 gives the results of the first five components (PC values) of the PCA, applied to the optimized simulated training data set. The specific absorption of CBC, adopted from the PROSPECT-PRO model, is indicated as black line. The three grey bars delineate the most important absorption features of lignin and cellulose (1730 nm, 2100 nm and 2300 nm). Since the first component (PC#1) carries more than 90% of the information, the PC value is rather equal (0.1) along the whole spectral range. The PCs #2 to #5, instead, show strong wavelength-dependent fluctuations. In the three specific spectral regions, all PCs, but in particular PC#4 and PC#5 follow a clear downward trend, pointing towards a signal of CBC.

### 3.3 Mapping Application Using PRISMA

**Figure 6:**
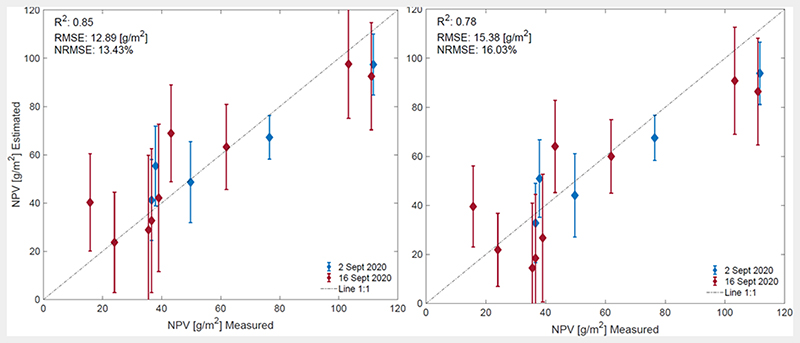
Measured vs estimated non-photosynthetic cropland biomass along 1:1-line including uncertainty intervals for EBD-optimized training data set (**left**), and EBD-optimized + 24 added non-vegetated (NV) spectra (**right**).

To ensure that the model can deal with a diversity of spectral signatures present within a full scene, the GPR algorithm was retrained after adding 24 NV spectra. Figure 6 compares the performances of the two NPV-GPR model versions: without and with NV spectra added. Including non-vegetated spectra to the training data set led to an acceptable lowering of R^2^ from 0.85 to 0.78 (RMSE: 12.9 vs. 15.4 [g/m^2^]). Uncertainty bars are also given, indicating the fidelity of the model.

**Figure 7:**
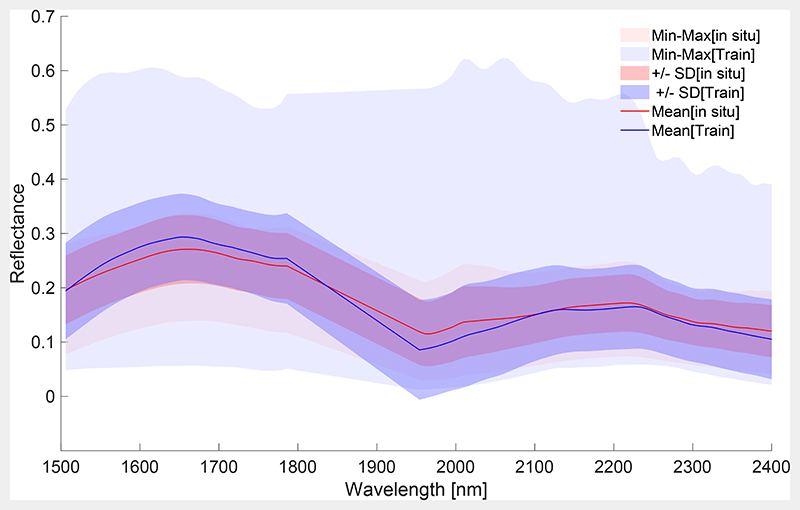
Illustration of total range of training versus in situ reflectances from the Slovakian campaign, with mean and SD. Training data base (Train) was simulated with PROSAIL-PRO and optimized with AL-EBD.

The descriptive statistics (i.e., standard deviations and ranges) of the final spectral training and in situ data sets are illustrated in Figure 7. It can be nicely seen that the measured spectra are well enclosed in the training spectra. By feeding a relatively broad range of training data into the algorithm, a sufficient degree of generalization can be ensured for building the final NPV-GPR model.

**Figure 8:**
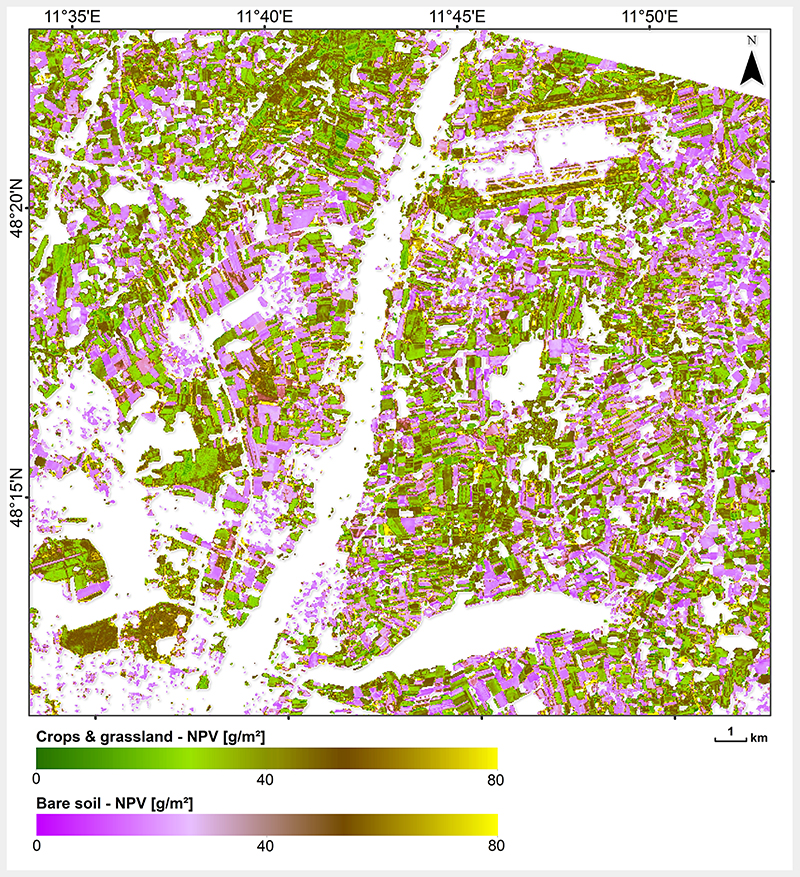
Mapping of non-photosynthetic cropland /NPV biomass using the full PRISMA scene covering the North of Munich, Germany. Based on image classification, only crops and grasslands, as well as bare soil areas were mapped; white zones were masked out.

**Figure 9:**
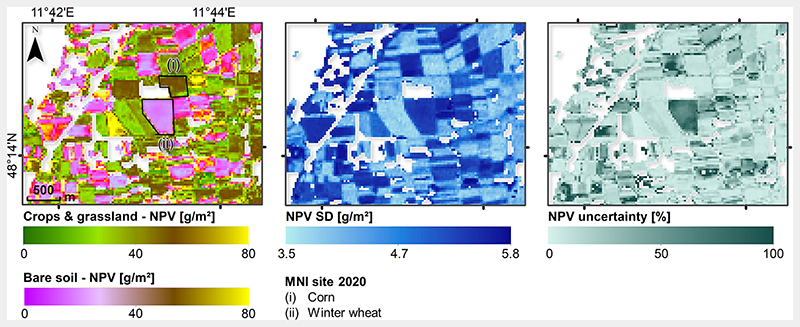
Zoom-in mapping example of non-photosynthetic cropland biomass estimation over long-term Munich-North-Isar test site. Left: non-photosynthetic cropland/NPV biomass estimates over crops and grasslands, and bare soils, middle: associated absolute uncertainties in form of standard deviation and right: relative uncertainty calculated with the coefficient of variation (CV). The two harvested MNI test fields of winter wheat and corn are also indicated. White zones were masked out based on image classification.

Subsequently, the mapping capability of the hybrid NPV-GPR model was tested through applying it on the PRISMA scene acquired in October 2020 over the North of Munich, Germany. The scene covered a wide range of surface types, including man-made such as a part of Munich, some smaller cities and villages. Natural vegetation is mainly present around the river Isar, which crosses the whole scene from South to North, with surrounding shrubs and forests. Besides the Isar, some other rivers and lakes are present. In order to exclude surface types not being considered during the model training phase, a simple classification was used for identifying the crops and grasslands, and bare soils areas (see Section 2.5). Though the majority of the area is characterized by intense agricultural usage [60], most of the fields have been harvested during this time of the year and only crop residues were left over. Additionally, grasses were a typical coverage, partly starting senescence. The resulting non-photosynthetic cropland biomass map is demonstrated in Figure 8, for which only crops and grasslands, as well as bare soil surfaces (with CR cover), were processed. In general, the map shows plausible estimates for the time of the year with non-photosynthetic biomass values between 60-80 g/m^2^. Since for this date no in situ reference data were available, mapping results can only be interpreted by plausibility. For a better interpretation, a subset over the agricultural test site MNI was processed (Figure 9). Two fields are indicated in the map, including one previously covered with corn and one with winter wheat. The latter was harvested more than two months before the PRISMA scene was acquired; thus, it was correctly classified as bare soil, and may be covered only by remaining CR. The corn site, classified as crop field, underwent harvesting approximately two weeks before the image acquisition. This caused a higher amount of CR (brown stalks) compared to the wheat field, as correctly recognized by the NPV-GPR model. As mentioned before, GPR models deliver information about the uncertainty along with the estimates. Hence, absolute (i.e., SD) and relative (coefficient of variation) uncertainties are shown over cropland and bare soil classes (Figure 9 middle and right). Here, we observe a lower uncertainty for the corn than for winter wheat field. This reflects the general trend of higher uncertainties over bare soil areas, suggesting a limited applicability of the NPV-GPR model to others than senescent cropped areas.

## 4 Discussion

In this study, we propose a hybrid strategy for retrieval of non-photosynthetic cropland biomass from imaging spectroscopy data. The workflow is composed of six steps ranging from (1) RTM simulations, over model optimization applying (2) active learning, (3) adding non-vegetated spectra to the training data set, (4) spectral dimensionality reduction, (5) classification of the image to identify land use of interest, and (6) applying the model to the PRISMA scene to estimate non-photosynthetic cropland biomass.

### 4.1 Active Learning and Spectral Dimensionality Reduction

A first key result is the substantially high accuracy achieved by the implemented AL strategy, despite the low number of available field in situ data. Thanks to the hybrid nature of the method, AL adapts the RTM simulated training data sets to real world situations by tuning them towards in field reference data. As also demonstrated by prior studies, this specific procedure with AL allows us to build robust and accurate retrieval models, which still retain independence and generality [61,70,78]. In our study, the EBD procedure achieved a reduction of the training data set to 25% from the full pool, thus eliminating noise and redundant information within the samples. The final light NPV-GPR retrieval model yielded excellent performances for non-photosynthetic cropland biomass estimation, being an optimal pre-requisite for processing large hyperspectral scenes. It must be further remarked that the implementation of AL replaces the need to add artificial noise to the spectral training data [60]. Second, PCA was chosen to reduce the high dimensionality (p) of the spectral data set. In principal, a PCA converts the data to a lower dimensional feature space by maximizing variance in q dimensions. On the one hand, this enhances processing efficiency and ensures a minimum of information loss on the other [73]. Although PCA gives the best possible representation of a p-dimensional data set in q dimensions, the new components only define linear functions of all p original spectral bands. Hence, it may be possible to interpret the first PCs—as they contain the majority of variance, and thus most of the information—but it may be more difficult to interpret higher components [73]. In our study, both simulated and measured NPV is composed of LAI and leaf carbon-based constituents. LAI is the major driver of canopy reflectance over the full spectral range (400-2500 nm), and is therefore the main contributor to PC#1. CBC, in contrast, leads to more subtle signals, exclusively due to some distinct absorption features in the SWIR. This subtle yet relevant information can only be contained in these higher components. The here used machine learning algorithm, GPR, gives relevance to them, though this comes with the risk that noise is also interpreted [60]. Generally, a high number of components (here we used 20) ensures that maximized variance of the created features (components) is captured by the NPV-GPR model. Moreover, reducing the spectral data to 20 components allows a fast image processing. One could, however, further investigate this number to identify the optimal trade-off between accuracy and runtime.

Another alternative can be feature or band selection, as successfully demonstrated by [52] for CNC retrieval. However, the impact of noise is probably better minimized when using feature transformation techniques, such as PCA, instead of single bands for model building. Besides, the information content of a generated component is supposed to be higher than of a single wavelength [32]. Nonetheless, the application of an automated GPR band analysis tool (GPR-BAT), as introduced by Verrelst et al. [89], could provide valuable information about the most informative spectral bands for NPV biomass estimation, being of interest, for instance, to design future satellites spectral channels. For such a task, however, more extensive field data sets would be required.

### 4.2 Mapping Performance of the NPV-GPR Model

With this work, we essentially present the first spaceborne non-photosynthetic cropland biomass map from space. Previous attempts rather focused on crop residue cover and were mainly based on Hyperion sensor data [90,91]. With the recent availability of PRISMA hyperspectral data, new opportunities open up to quantify landscape-scale non-photosynthetic cropland information, as recently demonstrated by Pepe et al. [21]. However, the low number of in situ reference data prevents from overall global validity of our model. Nonetheless, such data are still rarely available. The intense campaign carried out at the Slovakian site was labor-intensive as biomass was destructively sampled and carefully analyzed in the lab. Hence, these measurements can be considered as high quality data. Looking at the retrieved map (see Figure 9), the rather low NPV values over MNI site at the beginning of October seem realistic considering that mostly grassland was present and main crops have been harvested with only crop residues leftover on the fields. Moreover, the intra-field distributions are relatively narrow and spatially consistent, being an indirect measure for the accuracy of retrieval models [92]. Still, some remaining constraints have to be discussed here. The in situ soybean data set was sampled some weeks before and close to harvest, meaning it mainly represents very mature to fully senescent crop growth stages, but probably with lower biomass values than the typical cultivation of the area (corn and wheat). Therefore, mapping of crop residues may be well presented by most simulations, as also confirmed by relatively low uncertainties (see Figure 9). On the other hand, harvested or even ploughed fields with few remaining crop residues show a higher fraction of soil than vegetation. For these fields, less accurate retrievals may be obtained, confirmed by the higher uncertainties, as the NPV-GPR model was mainly adapted to learn spectral signatures of mature and senescent crop stages through the AL method. In general, the uncertainties given by GPR models provide information about the models' fidelity and could be used to identify spectra of surface types not considered in the training database [64]. Besides, knowledge about the phenological growth stages would support a correct interpretation of retrieval results generated by the NPV-GPR model.

### 4.3 Limitations and Future Challenges

The main bottleneck of our proposed method is the separation between green and nongreen vegetated elements by the applied RTM. The PROSAIL-PRO model is rather designed to simulate reflectance of photosynthetically active, thus green and alive vegetation. Still, by combining LAI with CBC, spectral effects of NPV can be simulated to some extent. With this, the model was solely trained for non-green vegetation, meaning that it likely performs unrealistic over green vegetated areas.

Hence, some adaptation should be made in a follow-up research. For instance, green and non-green vegetation types could be distinguished in a first step using the GBVI suggested by Delegido et al. [47] or comparable indices [93]. A similar approach was proposed by Amin et al. [25] through separate estimation of green and brown LAI. Moreover, alternative land use and cover classification methods could be applied on the scene, identifying a higher number of classes than in our study to reduce estimation uncertainty. Typically, machine learning classifiers are more successful when introducing more classes, thus with increasing complexity [94]. Additionally, a sufficient number of labeled data must be available. As the selected classes were easily visible on the original image, here over 200 samples were manually picked; yet more training samples may further improve the classification. Whilst the standard nearest neighbor method proved to be the most robust on the PRISMA scene, 17 other competitive supervised classifiers can be selected and tested with the ARTMO machine learning classification toolbox v1.00.

Alternatively, specific RTMs could be directly implemented, which take senescent structures and layers into account. Such strategies for NPV representation have been recently investigated. For instance, multi-layer structures of the SAIL model were developed, such as 2MSAIL [95] or senSCOPE [96], mimicking a variety of fractional and vertical gradients of NPV (senescence leaves) within the canopy. Multi-layer multi-organ RTMs such as 2MSAIL may allow a better representation of crop canopies via organ- and layer-specific parameterizations. In a future study, the 2MSAIL model should be investigated in respect to NPV or non-photosynthetic cropland biomass retrieval. However, it must be kept in mind that the higher number of input parameters provides an additional source of uncertainty. Furthermore, within a pixel, an explicit distinction between soil, photosynthetic and non-photosynthetic surface coverage could be made beforehand using spectral mixture analysis (SMA) [90]. In this way, different soil and residue types can be distinguished, which would minimize errors and increase the estimation accuracy.

The presented workflow for retrieving and mapping non-photosynthetic cropland biomass is currently under investigation within the framework of the planned operational CHIME mission. Along with other vegetation traits models, the NPV-GPR model could be implemented into CHIME's end-to-end (E2E) mission performance simulator [32]. In order to provide a trustful product retrieval chain when the mission is launched, the developed NPV-GPR model should be further improved in the next few years. This could be done, for instance, by exploiting the full spectral range with feature engineering methods. Moreover, the training database should be enlarged, including In Situ data from different crops, sites, and phenological growth stages, but also considering different tillage management practises, which may result in varying amounts of NPV on the soil surface. Ideally, PRISMA or other imaging spectroscopy scenes must be acquired over the same site where ground sampling takes place.

The application on PRISMA imaging spectroscopy data served perfectly as a benchmark since the sensor is a technology demonstrator providing the full spectral range required for NPV studies, and has proven to be suitable for scientific applications [85]. In principle, the non-photosynthetic cropland biomass models can be built for any type of imaging spectroscopy sensor data, as they will be provided by near-term missions (e.g., EnMAP, CHIME or SBG).

As a future vision, ideally these NPV-GPR algorithms could be implemented in cloud computing platforms for the generation of spatiotemporal continuous data streams [78,97]. In combination with greenness indicators, land surface phenology derived from nonphotosynthetic cropland biomass may help to improve the assessment and interpretation of seasonal changes, long-term trends or abrupt events in cultivated areas [98].

As a final remark, it must be emphasized that our retrieval model serves merely as a proof-of-concept to demonstrate that NPV biomass can be straightforwardly quantified. Yet, further improvements are still possible. For instance, within the ARTMO framework, various tuning options can be applied, e.g., processing of different field data, applying different parameter ranges and distributions for generating the training data base or exploring different dimensionality reduction and alternative AL methods. The software framework can be freely downloaded at http://artmotoolbox.com/ (accessed on 9 November 2021).

## 5 Conclusions

In this work, we present a hybrid workflow based on six steps for mapping nonphotosynthetic cropland or crop residue biomass from spaceborne imaging spectroscopy data. To achieve this, Gaussian process regression models were trained over a simulated data base by the PROSAIL-PRO model. The training data set included the carbon-based constituents variable at canopy level, which corresponds to lignocellulosic biomass, hence NPV. Further processing involved active learning methods to provide a representative training data set, and feature transformation in form of a PCA to remove noise and overcome spectral collinearity. In this respect, some higher PC values reflected specific absorption regions of lignin and cellulose. The final NPV-GPR model was successfully applied on a PRISMA hyperspectral image, providing estimates of non-photosynthetic biomass over croplands and bare soils, along with associated uncertainties. Moving ahead, the proposed retrieval strategy needs to be refined to enable the separation between green and non-green plants and crops. Furthermore, the collection of in situ reference data over multiple crop types in late mature and senescent growth stages is needed. As such, we can ensure the development of robust models by the time of the CHIME satellite's launch. We conclude that the suggested workflow presents a promising way towards mapping nonphotosynthetic cropland biomass by operational near-term imaging spectroscopy missions.

## Data Availability

Not applicable.
